# Administration of Exogenous Growth Hormone Is Associated with Changes in Plasma and Intracellular Mammary Amino Acid Profiles and Abundance of the Mammary Gland Amino Acid Transporter SLC3A2 in Mid-Lactation Dairy Cows

**DOI:** 10.1371/journal.pone.0134323

**Published:** 2015-07-30

**Authors:** Miriama Sciascia, David Pacheco, Susan A. McCoard

**Affiliations:** 1 Department of Nutritional Physiology ‘‘Oskar Kellner”, Leibniz Institute for Farm Animal Biology (FBN), Dummerstorf, Germany; 2 Animal Nutrition and Health Group, AgResearch Grasslands, Private Bag 11008, Palmerston North 4442, New Zealand; Auburn University, UNITED STATES

## Abstract

The objectives of this study were to (1) identify changes in plasma and mammary intracellular amino acid (AA) profiles in dairy cows treated with growth hormone (GH), and (2) evaluate the expression of mammary gland genes involved in the transport of AA identified in (1). Eight non-pregnant (*n* = 4 per group) lactating dairy cows were treated with a single subcutaneous injection of either a slow-release formulation of commercially available GH (Lactotropin 500 mg) or physiological saline solution. Six days after treatment, cows were milked and blood collected from the jugular vein for the analysis of free AA in the plasma. Cows were euthanized and mammary tissue harvested. Treatment with GH increased milk, protein, fat and lactose yields, with no effect on dry matter intake. Plasma concentrations of lysine and group I AA decreased significantly, and arginine, methionine, tyrosine and arginine-family AA tended to decrease in GH-treated cows. Concentrations of intracellular glycine, serine and glutamate increased significantly, with a trend for decreased arginine observed in the mammary gland of GH-treated cows. A trend for increased concentrations of intracellular total AA, NEAA and arginine-family AA were observed in the mammary gland of GH-treated cows. Variance in the concentration of plasma methionine, tyrosine, valine, alanine, ornithine, BCAA, EAA was significantly different between treatments. Variance in the concentration of intracellular lysine, valine, glutamine, EAA and group II was significantly different between treatments. AA changes were associated with increased mRNA abundance of the mammary gland AA transporter SLC3A2. We propose that these changes occur to support increased milk protein and fatty acid production in the mammary gland of GH-treated cows via potential mTOR pathway signaling.

## Introduction

Amino acids (AA) are used by tissues both as building blocks for protein synthesis and as signaling molecules to regulate the protein synthetic machinery [1]. During lactation the ruminant mammary gland must import from the plasma and endogenously synthesize sufficient quantities of AA to support cell turnover, epithelial cell differentiation and elevated milk protein synthesis [2]. Identifying and understanding changes in plasma and mammary gland intracellular AA concentrations and AA transport into the lactating mammary gland may provide fundamental knowledge towards the development of nutritional regimes aimed at elevating milk production.

Growth hormone (GH) is a well-established treatment model used by researchers to study mechanisms involved in the regulation of increased milk protein synthesis by the lactating ruminant mammary gland [3]. Treatment of ewes with exogenous GH can yield milk protein increases of 7% (mid-lactation Rahmani ewes) [4], and 12% (late-lactation Comisana ewes) [5]. Increased milk protein yield (42%) has also been observed in mid-lactation Alpine and Saanen goats treated with GH [6]. Holstein cows treated with exogenous GH in early and late-lactation can increase protein yield by 14% and 18%, respectively [7], 11% in mid-lactation Friesian cows [8] and 30% in mid-lactation Jersey cows [9]. The mammary glands of cows treated with GH elevate arterio-venous extraction of a range of AA from the blood to support increased milk protein production [10].

Specific transporter families situated in the plasma membrane surface of mammary epithelial cells (MEC) mediate the extraction of AA from blood [11]. Studies from a range of species [12], including bovine [11] indicate that the mammary gland may contain members of every known AA transporter family and that the expression of these transporters is regulated by stage of lactation, metabolic need, nutritional supply and specific hormonal changes [13].

The nutrient-sensing mechanistic target of rapamycin (mTOR) pathway is known to link AA sufficiency, hormonal signaling and AA transporter gene expression [14]. The core of the mTOR pathway is the mTOR protein, which exists in two complexes: mTORC1 and mTORC2 [15]. Each complex plays a role in the regulation of protein synthesis with mTORC1 playing the major function via direct regulation of cellular machinery involved in nucleocytoplasmic export of growth-promoting mRNA and protein synthesis [15]. We have previously reported that increased abundance of mTOR and phosphorylated mTOR (Ser2448) in the mammary glands of cows treated with exogenous GH occurred independently of GH induced IGF-1 signaling [16]. *In vitro* and *in vivo* studies show that activation of mTOR signaling can be stimulated by AA sufficiency and/or specific AA accumulation [17, 18]. The precise role of individual AA transporters is yet to be fully elucidated; however the expression of a number of AA transporter genes has been linked to mTORC1 activation. Increased expression of the L-glutamine transporter solute carrier (SLC)1A5 elevates mTORC1 activation in both human hepatoma cells [19] and the mammary epithelial cell line MCF-7 [14], via interactions with the heterodimeric L-leucine / L-arginine transporter SLC3A2/SLC7A5 [14].

The aims of this study were to (1) identify changes in plasma and mammary gland intracellular AA during increased milk protein synthesis observed in dairy cows treated with GH, and (2) evaluate the expression of mammary gland genes involved in the transport and utilization of AA identified in (1).

## Materials and Methods

### Animals and Treatments

All procedures involving animals were carried out in strict accordance with the 1999 Animal Welfare Act of New Zealand. The protocol was approved by the AgResearch Grasslands Animal Ethics Committee (Palmerston North, New Zealand; Permit number: 49/02). The trial design and sample collection methodology has been described in detail elsewhere [9]. Eight non-pregnant, lactating second parity Jersey cows (178 to 200 days postpartum) were housed in separate indoor stalls and fed a diet formulated to exceed requirements for metabolizable energy (ME), protein and essential AA (Table 1). Dry matter intakes for each cow were averaged for the experimental period (d 0 to d 6). Cows were fed daily at 0700, 1600, 2000 h and had *ad libitum* access to fresh water. Milking was performed at 0730 and 1630 h, milk yield throughout the experimental period was recorded, and samples were collected at each milking for measurement of milk composition. The concentration of milk protein, fat and lactose was measured by infrared spectroscopy by the milk analysis laboratory of Livestock Improvement Corp. (Hamilton, New Zealand). A single subcutaneous injection of either a slow-release formulation of commercially available GH (Lactotropin) 500 mg or saline solution was administered to four cows in each group. Six days following injection, blood was collected from the jugular vein of each cow for the analysis of plasma free amino acids (FAA).

**Table 1:** Diet ingredients (% of ingredient in the dry matter) and nutritional composition (% in the dry matter, unless otherwise stated).

Item	
Pasture silage	71.43
Barley	9.70
Wheat	9.71
Soya bean meal (48% CP)	7.14
Molasses	1.14
Limestone (33% calcium)	0.29
Dicalcium phosphate	0.29
Calcined magnesium	0.14
Sodium chloride	0.11
^1^Hi Spec Dairy Premix	0.04
^2^Sel-Plex	0.01
Composition	
DM%	48.43
OM, % of DM	89.4
CP, % of DM	18.53
ADF, % of DM	24.2
NDF, % DM	36.9
NFC, % of DM	35.5
ME (MJ per kg DM)	23.0
^3^NEl (MJ per kg DM)	6.95
^3^MP (g / day)	908

^1^Hi Spec Dairy Premix (per 2.5 g: 350 mg Zinc, 120 mg Copper, 320 mg Manganese, 2 mg Cobalt, 3 mg Selenium, 4 mg Iodine, 20,000 iu Vitamin A, 2,000 iu Vitamin D_2_, 50 mg Vitamin E).

^2^Sel-Plex–proprietary organic form of selenium yeast.

^3^The estimates of NEl and MP were obtained using the NRC Dairy Cattle (2001) software, including the composition of the ingredients as described in Table 1.

Cows were euthanized using an overdose of sodium pentobarbitone (Provet NZ, Auckland, New Zealand), and all efforts were made to minimize suffering. Mammary parenchymal tissue (alveolar tissue free of large ducts and blood vessels) collected, snap frozen in liquid nitrogen and stored at -80°C within 5 min of slaughter, for subsequent analyses.

### Free Amino Acid Profiles

Mammary tissue and plasma FAA were analyzed using High-Performance Liquid Chromatography (HPLC), as previously described in [20] and [21] and reported as μmol per gram of mammary tissue or per liter of plasma.

#### Tissue.

A 200 mg sample of frozen (-80°C) mammary tissue was homogenized in 1.75 ml of Seraprep (Pickering Laboratories, Alphatech Systems Ltd, Auckland, New Zealand) and 20 μL of aminoguanidinopropionic acid (25 μM / mL) added as an internal standard. Samples were left on ice for 20 min, and 40 μL of 5.88 M lithium hydroxide buffer added, followed by centrifugation at 13,680 *g* for 5 min.

#### Plasma.

A 500 μL sample of blood plasma was mixed with 500 μL lithium extraction buffer (14 g/L lithium chloride, 3 g/L lithium hydroxide, 1 g/L phenol, 50 g/L sulfosalic acid) and placed on ice for 15 to 20 min. Volumes of 10 μL lithium hydroxide (5.88 M) were added to the sample to obtain a final pH of 1.5 to 2, followed by centrifugation at 13,680 *g* for 5 min at 4°C.

#### Tissue and Plasma.

The resulting supernatant was filtered through a 4 mm 0.2 μL syringe filter. The filtered sample was analyzed for FAA using a Shimadzu LC10Ai HPLC (Shimadzu Oceania Ltd., Auckland, New Zealand), fitted with a high-efficiency lithium-ion exchange column (3 mm i.d. × 150 mm; Pickering Laboratories, Shimadzu Oceania Ltd.). Injected volumes were 10 μL, with a reagent flow rate of 0.3 mL / min and a run time of 162 min between injections, using Li buffers as eluents and ninhydrin post-column derivatisation. The minimum FAA detection range for this method is 0.14 to 0.51 pmol, as assessed using a mineral acid casein control hydrolyzed at 110°C for 22 h in sealed evacuated tubes.

### Quantitative Real-Time PCR

#### Primer Design.

Primers were designed against *Bos taurus* and *Ovis aries* mRNA sequences to allow them to be used across a range of projects involving both species. Publicly available mRNA sequence data from NCBI (www.ncbi.nlm.nih.gov) and CSIRO (www.livestockgenomics.csiro.au/sheep/) were used in conjunction with the Roche Universal ProbeLibrary assay design centre (www.roche-applied-science.com/sis/rtpcr), as previously described [16]. Primers were made by Integrated DNA Technologies and purified using desalting to remove short truncated products and small organic contaminants (IDT, Antwerp, Belgium). Primer sequences are presented in Table 2.

**Table 2:** Name, symbol, accession number, primer sequence and amplicon size of genes analysed by qPCR.

Gene Name	Symbol	Accession number	Hybridization	Primers (5′ to 3′)	Amplicon Size (bp)
**Reference Genes**					
Mitogen-activated protein kinase 1	MAPK1	NM_175793.2	Forward Reverse	TCGCAGGAAGACCTGAATTG TCCTCTTGTGAGGGTTGAACG	165
(Src homology 2 domain) transforming protein 1	SHC1	NM_001164061.1 NM_001075305.2	Forward Reverse	CAGTCCATCTCGTTTGCATC GGCTCTTCCTCCTCCTCATC	260
**Amino Acid Transporters**					
Solute carrier family 1, member 1	SLC1A1	NM_174599.2	Forward Reverse	TCTGGTGGATTTCTTCAATGCT TAAAGGCCCAGTTTGCGG	151
Solute carrier family 1, member 5	SLC1A5	NM_174601.2	Forward Reverse	AGGAGAGATTGTTCAACGGC GAAGAAGCGAATGAGCAGC	152
Solute carrier family 3, member 2	SLC3A2	NM_001024488.2	Forward Reverse	AGCTGAGTGGCAGAACAT TTAAGCTGGAGTGTGACAGGTA	152
Solute carrier family 6, member 14	SLC6A14	NM_001098461.1	Forward Reverse	CTGATTGACCACTTCTGTGC GCAAGTTCTCCACCATAGCC	161
Solute carrier family 7, member 5	SLC7A5	NM_174613.2	Forward Reverse	TCTGCAGCACAGCGTTC GCTTTAAGGCAACGAGACGTA	154

#### RNA Extraction and cDNA Synthesis.

RNeasy minikits, with on-column DNase I treatment (Qiagen, San Diego, CA, USA) were used to purify total RNA. Purified RNA was quantified using a NanoDrop Spectrophotometer (ND-1000; NanoDrop Technologies), and RNA quality assessed by running 1 μg on a 1% non-denaturing agarose gel, stained with SYBR Safe (Invitrogen, Auckland, New Zealand). The SuperScript VILO cDNA Synthesis Kit Total was used to reverse transcribe 500 ng of purified RNA using the manufacturers modified protocol of 120 min at 42°C (Invitrogen). Quantitative real time PCR was performed as previously described by Sciascia et al [16].

### Statistical Analysis

#### Dry matter intake.

The TTEST procedure in SAS 9.1 (SAS Institute Inc., Cary, North Carolina, USA) was used to analyze the data. Probability values ≤ 0.05 were considered statistically significant.

#### Milk and composition yields.

Data were analyzed using the Mixed procedure in SAS Version 9.3 (SAS Institute Inc., Cary, North Carolina, USA). Measurements made before GH was administered (i.e., d 0) were used as covariates for the respective variable of interest. A linear model, including the covariate and the fixed effects of treatment, day and the interaction of treatment by day was used for the analysis. Repeated measurements made on each cow were accounted for using the repeated statement with day as effect and cow within treatment as the subject for the repeated measurements, as per Littell et al [22], with cow within treatment as random effect. After fitting several variance-covariance structures to the repeated measurement data, a first-order autoregressive matrix was deemed the best fit for the covariance structure of the repeated measurements, as assessed by the Bayesian and Akaike’s information criteria. Data are presented as least squares means and standard errors of the mean (SEM). Probability values ≤ 0.05 were considered statistically significant, and values > 0.05 but ≤ 0.15 are discussed as trends.

#### Amino acid profiles.

Differences between GH-treated and control animals were assessed via two-sample *t*-tests, which included the fixed effects of animal, treatment and amino acid (individual or pool), for individual AA, total-AA and the sums of essential amino acids (EAA), non-essential AA (NEAA), branched-chain AA (BCAA), the arginine-family AA (arginine, aspartate, asparagine, glutamate, glutamine, proline, ornithine) and the groups of AA defined by their mammary metabolism as outlines by Mepham [23]: group I (methionine, phenylalanine, tyrosine) and group II (arginine, histidine, isoleucine, leucine, lysine, valine) using the TTEST procedure in SAS 9.1 (SAS Institute Inc., Cary, North Carolina, USA). Initial data exploration indicated that for some AA the variances were not homogenous between treatments. Thus, the analysis was done using the Cochran approximation to adjust the probability values for those AA with unequal variance per treatment. Both the probabilities for the null hypotheses of equal mean and equal variance are reported. Probability values ≤ 0.05 were considered statistically significant, whilst values ≤ 0.15 were considered a trend.

#### qPCR Assay.

Two reference genes, mitogen activated protein kinase 1 (*MAPK1*) and Src homology 2 domain containing 1 (*SHC1*) were identified using NormFinder (http://www.mdl.dk/publicationsnormfinder.htm; [24]). Reactions were performed in triplicate with all data entered into the relative expression software tool (REST) 2009 (http://www.gene-quantification.de/rest-2009.html; [25]) and the fold change in expression ratios between the means of two treatment groups determined. The PCR efficiency and quantification cycle values were obtained for each sample using LinRegPCR [26] and all qPCR data reported as per published guidelines [27]. Probability values ≤ 0.05 were considered statistically significant.

## Results

### Effect of GH on Dry Matter Intake and Milk Yield / Composition

**Fig 1:**
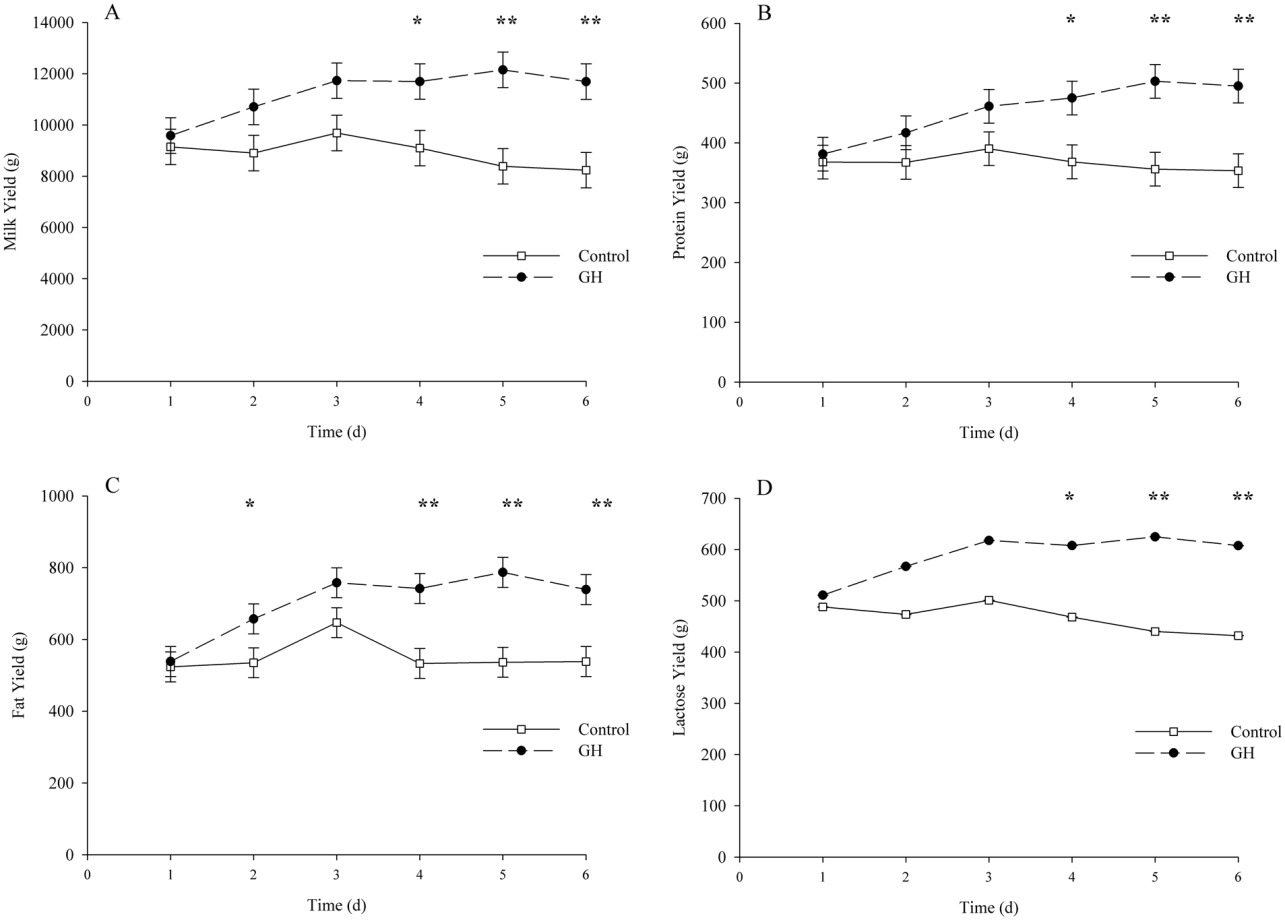
Milk, protein, fat and lactose yields of lactating dairy cows treated with growth hormone. Growth hormone (GH) treatment significantly increased milk (A), protein (B), fat (C) and lactose (D) yields from d 4 to d 6, compared to controls (*n* = 4 per treatment). To assess differences between treatment groups a linear model, including fixed effects of treatment and day effects and their interaction was used. Measurements collected daily over the experiment were treated as repeated measurements using cows within treatment as the subjects, as per Littell et al [22]. The figures show least squares means; Asterisks denote significant differences between treatments (*: *P* < 0.05); (**: *P* < 0.01). Error bars denote the standard error of the mean. Milk and protein yield data adapted from Hayashi et al (2009).

Significant differences in milk, protein, fat and lactose (Fig 1A to 1D) yields were observed between GH and control cows by d 4 of GH treatment. Six days post GH administration, when cows were euthanized and the tissue (blood, mammary) samples collected, milk, milk protein, milk fat and lactose yields were 1.42, 1.40, 1.37 and 1.41 times greater in the GH-treated animals, compared with controls. No difference was observed in dry matter intake between GH-treated and control cows (16.45 ± 0.91 vs. 14.57 ± 1; P = 0.21).

### Effect of GH treatment on absolute concentrations of individual plasma and intracellular mammary tissue free amino acids

**Fig 2:**
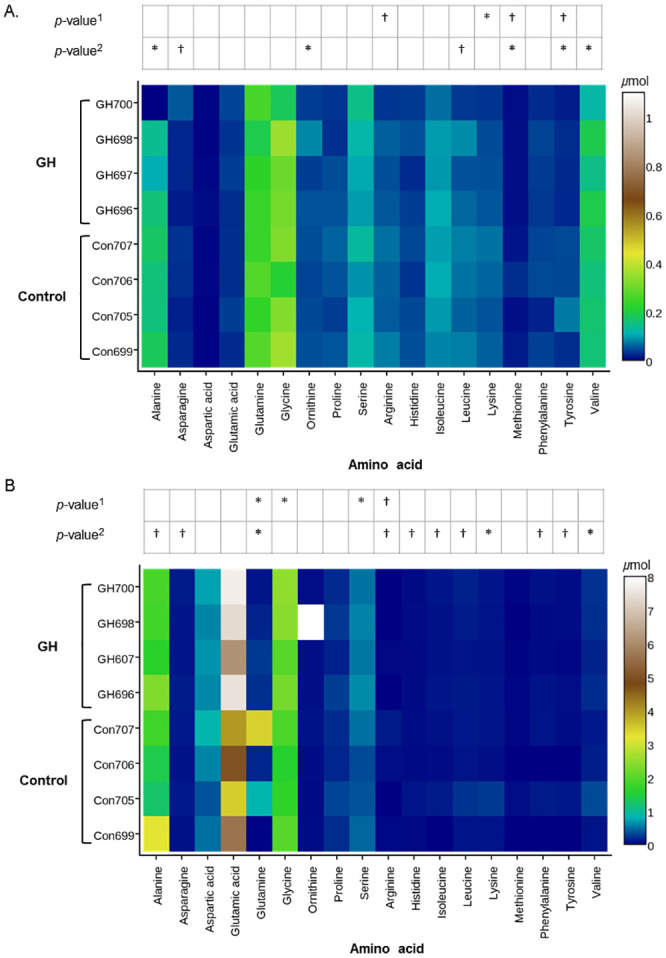
Growth hormone treatment changes individual plasma and mammary tissue amino acid concentrations and variance. Absolute concentrations (heatmap) and statistical significance of differences in the mean concentration^1^ and variance^2^ of amino acids in the plasma (A) and the mammary tissue (B) of cows treated with growth hormone (GH) or saline solution (Control) (*n* = 4 per treatment). Absolute concentration of plasma lysine was decreased in GH-treated cows with a similar trend observed for arginine, methionine and tyrosine (A). Absolute concentrations of other individual plasma AA were unaffected (A). The absolute concentration of the arginine was decreased in mammary tissue of GH-treated cows compared to controls (B). A significant increase in the absolute concentration of glutamine, serine and glycine was also observed in the mammary tissue of GH-treated cows (B). Reduced variance in the concentration of plasma AAs methionine, tyrosine, alanine, ornithine and valine with a similar trend for leucine and asparagine (A) and mammary tissue AAs glutamine, lysine and valine with a similar trend for alanine, asparagine, phenylalanine, tyrosine, arginine, histidine, leucine and isoleucine (B) was observed in GH-treated cows. Differences were assessed using the TTEST procedure in SAS 9.1 (SAS Institute Inc., Cary, North Carolina, USA) which included the fixed effects of animal, treatment and amino acid (individual or pool). *: *P* ≤ 0.05; and trend: †: *P* ≤ 0.15.

In the plasma, a significant decrease in the absolute concentration of lysine (*P* = 0.04) and trends for decreased concentration of arginine (*P* = 0.06), tyrosine (*P* = 0.09) and methionine (*P* = 0.14) were observed in GH-treated compared to control cows (Fig 2A). No effect was observed in the concentration of other individual plasma AA (Fig 2A). In mammary tissue, a trend for decreased arginine (*P* = 0.10) concentration was observed in GH-treated compared to control cows (Fig 2B). A significant decrease in glutamate (*P* < 0.01), serine (*P* = 0.02) and glycine (*P* = 0.02) concentrations were observed in GH-treated compared to control cows (Fig 2B).

### Effect of GH treatment on the concentration of different groups of plasma and intracellular mammary tissue amino acids

**Fig 3:**
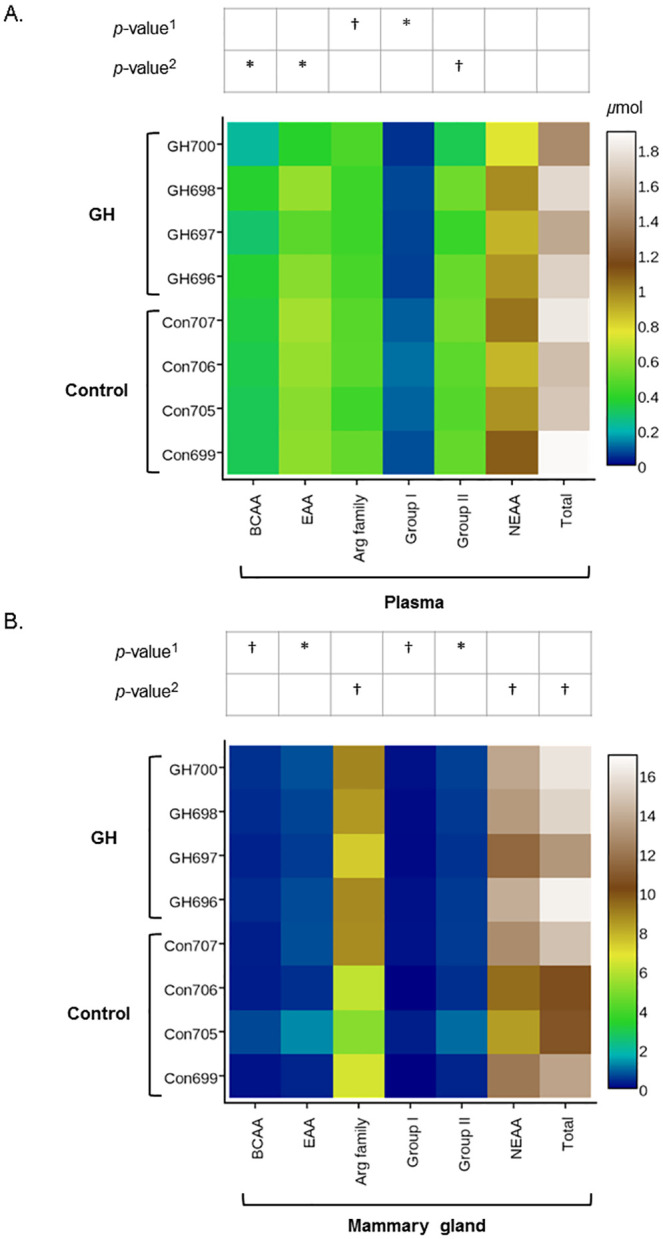
Growth hormone treatment changes plasma and mammary tissue amino acid group concentration and variance. Absolute concentrations (heatmap) and statistical significance of differences in the mean concentration^1^ and variance^2^ of amino acid groups in the plasma (A) and the mammary tissue (B) of cows treated with growth hormone (GH) or saline solution (Control) (*n* = 4 per treatment). In plasma, there was a significantly reduction in group I AA and a trend for reduced arginine-family AA in GH-treated compared to control cows. In mammary tissue there was a trend for increased concentrations of NEAA, total AA and arginine-family AA in GH-treated compared to control cows. The variance in plasma concentrations of BCAA, EAA were reduced, while a similar trend was observed for group II. In the mammary tissue, the variance in concentration for BCAA, EAA, group I and group II was also reduced. Differences were assessed using the TTEST procedure in SAS 9.1 (SAS Institute Inc., Cary, North Carolina, USA) which included the fixed effects of animal, treatment and amino acid (individual or pool). *: *P* ≤ 0.05; and trend: †: *P* ≤ 0.15).

In the plasma, concentration of group I AA were significantly reduced (*P* = 0.01), while a trend (*P* = 0.11) in reduced concentration of arginine-family of AA was observed in GH-treated cows compared to controls (Fig 3A). No difference was observed for the concentrations of EAA, NEAA, total AA, group II or BCAA (Fig 3A). In the mammary tissue, there were trends for increased concentrations of NEAA (*P* = 0.09), total AA (*P* = 0.06) and the arginine-family of AA in GH-treated cows compared to controls (Fig 3B). No difference was observed for the concentrations of EAA, group I, group II or BCAA (Fig 3B).

### Effect of GH treatment on the variance of individual plasma and intracellular mammary tissue free amino acids

The variance of the concentrations of plasma methionine, tyrosine, valine, alanine, and ornithine was significantly reduced, whilst trends for reduced variance of the concentrations of leucine and asparagine were observed in GH-treated compared to control cows (Fig 2A). In the mammary tissue, variance of the concentrations of lysine, valine and glutamine was significantly reduced, whilst a trend for reduced variance of the concentrations of arginine, histidine, isoleucine, leucine, phenylalanine, tyrosine, alanine and aspartate was observed in GH-treated compared to control cows (Fig 2B).

### Effect of GH treatment on the variance of plasma and intracellular mammary tissue free amino acids groups

In the plasma the variance in concentration of the EAA and BCAA groups was significantly reduced, while a trend for reduced variance in the concentration of group II AA was also observed (Fig 3A). No effect was observed in the variance in concentration of NEAA, arginine-family, group I and total AA (Fig 3A). The variance of the concentrations of intracellular mammary EAA and group II were significantly reduced, while a trend for reduced variance in the concentration of BCAA and group I were also observed (Fig 3B). No effect was observed in the variance in concentration of NEAA, arginine-family, NEAA and total AA (Fig 3B).

### Effect of GH treatment on amino acid transporter gene expression

**Fig 4:**
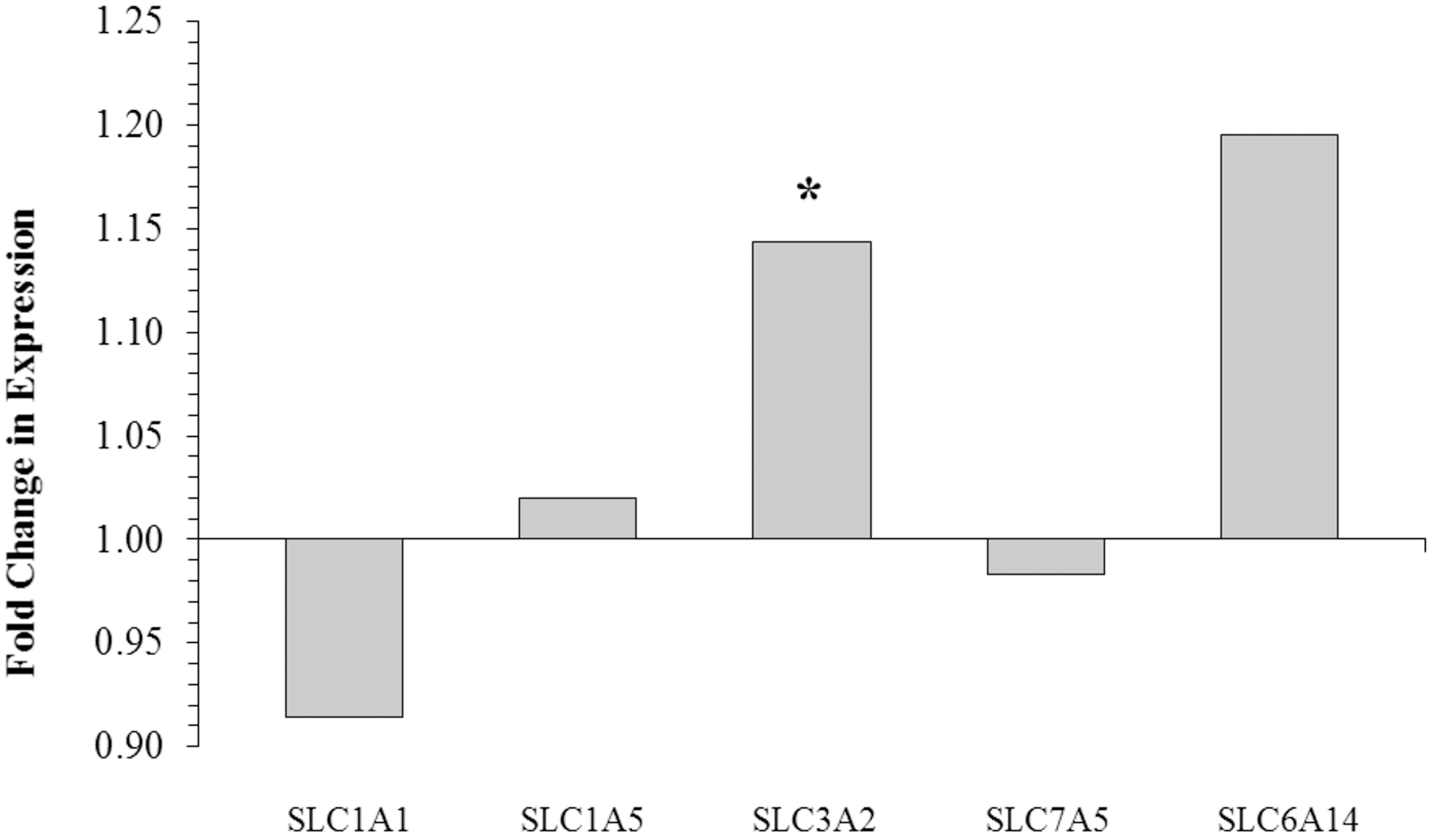
Mammary gland amino acid transporter mRNA abundance in dairy cows treated with growth hormone. Treatment with GH increased the mRNA abundance of heterodimeric heavy-chain L-arginine transporter SLC3A2. No significant change in mRNA abundance was detected for any of the other transporters measured. Mitogen-activated protein kinase 1 (MAPK1) and Src homology 2 domain containing 1 (SHC1) had the most stable expression of all the genes analyzed using Normfinder and were used as reference genes. The figure shows the fold change in gene expression in GH-treated cows compared with control cows as assessed by the relative expression software tool (*n* = 4 per treatment), *: *P* ≤ 0.05.

Treatment with GH increased mRNA abundance of the amino acid transporter SLC3A2 by 1.15-fold (Fig 4). The abundance of SLC1A1, SLC1A5, SLC7A5 and SLC6A14 mRNA did not change significantly.

## Discussion

This study utilized the well-established GH-treatment model to study increased milk production in lactating dairy cows. Results showed cows at d 6 post GH-treatment had increased yields of milk, protein, fat and lactose with no observable difference in dry matter intake. In addition, previously published work by our group showed GH-treatment has no observable effect on milk composition [9]. These results suggest that GH-treatment may increase milk production by repartitioning nutrients to the mammary gland to support equally the production of milk protein, fat and lactose.

Milk proteins in the lactating ruminant mammary gland are primarily synthesized from circulating plasma AA, which can be classed into three groups based on their requirement for supporting milk protein synthesis, group I, group II and NEAA [10]. The group 1 AA are transferred from plasma to milk protein in a ratio close to 1:1 [10]. We observed a decrease in the concentration of group I AA in plasma, but no change in the mammary tissue intracellular concentration. Of the individual AA that comprise group I, only plasma methionine and tyrosine concentrations were reduced in GH-treated cows, with no effect observed in mammary tissue intracellular concentrations. These results indicate that the mammary gland of GH-treated cows may have increased uptake of the group I AA to support the observed increase in milk protein synthesis. In addition, phenylalanine concentrations may not have changed in plasma or mammary tissue of GH-treated cows, as mammary requirements for tyrosine may have been met from plasma uptake rather than synthesis from phenylalanine [23].

In this study the plasma and mammary tissue intracellular concentrations of group II AA and BCAA were unaffected by GH-treatment, whilst plasma lysine and arginine, and mammary tissue intracellular arginine concentrations were decreased. Lysine and arginine belong to the group II AA which are extracted from plasma in greater quantities than those found in milk protein, which suggests they participate in other biological processes to support increased milk production [28]. A considerable amount (20 to 35%) of lysine taken up by the lactating ruminant mammary gland is oxidized [29]. In mammalian cells the oxidative metabolism of lysine ends in the production of acetyl-CoA, an essential precursor of fatty acid synthesis and component of the Krebs’s cycle production of NEAA [30]. In another study by our group, (McCoard, *submitted*) microarray and qPCR validation data collected from the same mammary tissue, shows expression of two key enzymes involved in converting acetyl-CoA into fatty acid precursors, FABP3 [31] and FADS2 [32] were increased in the mammary glands of GH-treated cows. In the current study, intracellular concentrations of glutamate, a by-product of lysine metabolism to acetyl-CoA [33] were elevated in response to GH, and expression of the primary glutamate AA transporter SLC1A1 and plasma glutamate concentrations were unaffected. These results suggest that the mammary glands of GH-treated cows increase the extraction of plasma lysine to maintain an intracellular supply of acetyl-CoA, which could be utilized to support, increased the observed increase in milk fat production, and as a by-product, elevate intracellular glutamate concentrations.

The NEAA are generally not taken up from plasma in sufficient quantities to account for their secretion in milk [23]. It has been proposed by Bequette [34] that the lactating mammary gland catabolizes EAA to provide carbon and nitrogen for NEAA synthesis. Our results support this observation as the levels of individual and pooled plasma NEAA were unaffected by GH-treatment, but the NEAA concentration in mammary tissue was increased. The concentrations of the NEAA serine and glycine were also increased in response to GH. Analysis of microarray and qPCR validation data collected from another study by our group, using the same mammary tissue, shows that expression of the glycolytic pathway genes phospho-glycerate kinase 1 (PGK1) and glucose-6-phosphate isomerase (GPI) were increased in the mammary glands of cows treated with GH (McCoard et al, *submitted*). A function of glycolysis in the lactating ruminant mammary gland is the production of pyruvate, which is used as a carbon skeleton for the synthesis of AA via the Krebs cycle. The ruminant mammary gland synthesizes serine using pathway intermediates of glycolysis (3-phosphoglycerate) and the Krebs cycle (α-ketoglutarate), whilst glycine is directly synthesized from serine [35]. Interestingly, PGK1 catalyzes the glycolytic production of 3-phosphoglycerate, the direct precursor of serine biosynthesis. These observations suggest that the mammary glands of GH-treated cows increase glycolysis to provide carbon skeletons for the production of AA via the Krebs cycle, and the glycolytic pathway precursor 3-phosphoglycerate for the production of serine and glycine.

In this study we show the arginine-family of AA decreased in plasma and increased in the mammary tissue of GH-treated cows. These results suggest that the mammary glands of GH-treated cows may increase the uptake of arginine-family AA to support increased milk production. Individually, the only arginine-family of AA to change was glutamate and arginine, of which arginine decreased in both plasma and mammary tissue. We earlier proposed that the increased concentration of mammary glutamine was a result of lysine metabolism. Arginine may play a more critical role as it has the highest uptake relative to milk protein output (200 to 300% [23]), and is utilized by lactating mammary tissue to produce nitric oxide [36], synthesize proline, an AA which is not taken up in sufficient quantities from circulating plasma to support both milk protein synthesis and polyamine synthesis [37] and regulate protein synthesis via mTOR pathway regulation [14]. While future studies need to be conducted to identify changes in mammary genes involved in arginine metabolism, we have investigated the main transporters involved in arginine uptake. In this study, we show that in response to GH-treatment expression of the AAT SLC3A2, which co-transports plasma arginine into cells, is increased. SLC3A2 forms a heterodimeric complex with several smaller subunits that change the specificity of the AA being imported, it also has a reciprocal regulatory connection with mTOR [14]. Studies show that SLC3A2 activity can be driven by SLC1A5 import of glutamine, which is rapidly effluxed by SLC3A2 when complexed with SLC7A5 [14]. In this study, the expression of SLC7A5 and SLC1A5 was unaffected by GH treatment, indicating other regulatory partners of SLC3A2 may be involved in arginine uptake.

In this study, treatment with GH was associated with reductions in the variance in concentration of plasma and mammary tissue EAA, eight out of the nine individual EAA in mammary tissue and four in plasma. We propose that the reduced variance in the concentration of EAA is an outcome of orchestrated events in the lactating gland to optimize AA supply relative to protein synthetic activity in response to GH. Associated with GH-treatment was a reduction in the variance in the concentration of the BCAA pool in the plasma and mammary tissue, all three individual BCAA in mammary tissue and two in plasma. The BCAA have been shown in several studies to activate the mTOR pathway [15, 17], which we have previously shown to be activated in the mammary tissue used in this study [16]. It is also interesting to note that in the model proposed by Bequette et al [34] the excess amino groups formed as by-products of NEAA synthesis from EAA are ultimately transaminated to form alanine, aspartate or glutamine, the three mammary NEAA whose variance in concentration was reduced in this study.

## Conclusion

In summary, we show that increased milk, lactose, fat and protein yields in lactating dairy cows treated with GH is associated with changes in the concentration and variance in concentration of AA in plasma and mammary tissue, and increased mRNA abundance of the mammary AAT SLC3A2. Further investigation will be required to elucidate the potential role SLC3A2 and the changes in AA concentrations play in regulating milk production in GH-treated cows.
